# Do stress symptoms impact handgrip strength and firearm shooting accuracy among military police officers?

**DOI:** 10.3389/fpsyg.2025.1557524

**Published:** 2025-03-27

**Authors:** Valter R. Vasconcelos Junior, Romulo C. T. Costa, Geanderson S. Oliveira, Pedro F. C. Fortes Junior, Alexandre F. Machado, Roberta Luksevicius Rica, Gregg S. Mallett, Valentina Bullo, Marco Bergamin, Stefano Gobbo, Danilo Sales Bocalini

**Affiliations:** ^1^Military Police of Espirito Santo, Vitoria, Brazil; ^2^Experimental Physiology and Biochemistry Laboratory, Center of Physical Education and Sport, Federal University of Espírito Santo, Vitoria, Brazil; ^3^Physical Education, Estacio de Sá University, Vitoria, Brazil; ^4^Department of Kinesiology, Health Promotion, and Recreation, University of North Texas, Denton, TX, United States; ^5^Department of Medicine, University of Padova, Padova, Italy

**Keywords:** physical activity, handgrip strength, stress, police force, firearm

## Abstract

**Introduction:**

The operations carried out by the Military Police (MP) in public safety and violence prevention, driven by the evolving needs of contemporary society, impose specific physical and psychological requirements on their personnel that are typically not encountered by the general population. In this context, factors such as exposure to violence, frequent risk of death, heavy workloads, challenging working conditions, and elevated stress levels associated with their missions are integral to the daily experiences of MP officers, rendering them one of the most vulnerable professional groups. Therefore, the objective of this study was to assess whether the presence of stress symptoms impacts handgrip strength and firearm shooting accuracy among MP officers.

**Methods:**

The study included 24 third-year officer cadets stationed at the Espírito Santo Military Police Academy; all participants volunteered. Stress levels were measured using Lipp’s Inventory of Stress Symptoms (LSSI), categorizing participants into two groups: those exhibiting stress symptoms (CE, *n* = 11) and those without (SE, *n* = 13). Firearm shooting was conducted during the basic training track evaluation of Defensive Shooting in Life Preservation, employing the Giraldi Method, with shots directed at a fixed target 5 meters away. Key metrics evaluated included the time taken to execute the shots (T), total score (TS), and shooting accuracy calculated as (50 x TS/T). Handgrip strength (HGS) was measured in the dominant hand (DH), non-dominant hand (NDH), and shooting position (SP) using a handgrip dynamometer.

**Results:**

The average age of participants was 31.13 ± 2.49 years, with an average length of service of 7.71 ± 3.75 years and an average body mass index (BMI) of 25.58 ± 2.45 kg/m^2^. No significant differences (*p* > 0.05) were observed between groups concerning the variables T (CE: 63.45 ± 9.09, SE: 65.69 ± 9.09), TS (CE: 9.36 ± 1.03, SE: 9.38 ± 1.04), TS/T (CE: 7.47 ± 1.11, SE: 7.25 ± 1.14), DH (CE: 35.66 ± 8.29, SE: 38.54 ± 6.88), and NDH (CE: 34.19 ± 6.48, SE: 37.39 ± 8.56). However, significant differences (*p* < 0.05) were identified between the groups regarding the SP parameter (CE: 39.96 ± 10.40, SE: 47.06 ± 8.57).

**Discussion:**

The findings of this study indicate that, although the presence of stress symptoms led to variations in HGS during shooting position, it did not significantly impact shooting accuracy among MP cadets.

## 1 Introduction

The duties performed by the Military Police (MP) in public safety and violence prevention are progressively shaped by the dynamic needs of contemporary society, imposing distinct physical and psychological demands on their personnel that are typically faced by the public. Within this framework, factors such as continuous exposure to violence, consistent threats to life, high workloads, harsh working conditions, and increased stress levels inherent with their responsibilities are central to the everyday lives of MP officers (Marinho et al., 2018). These conditions make this group one of the most vulnerable in their professional practice (Marinho et al., 2018; Souza and Minayo, 2005).

In this context, studies presented in the Brazilian Public Security Yearbook (IPEA/FBSP, 2024) indicate that the incidence of police fatalities during service has reached alarming levels. For instance, a study conducted by Bueno et al. (2013) reveals that the homicide rate among police officers in Brazil is three times higher than that of the general population. Similarly, research by Vaz (2023), which compiled data on both military and civil police fatalities from Public Security Yearbooks between 2019 and 2023, highlighted that from 2018 to 2022, 1,009 on-duty or off-duty officers fell victim to homicide in Brazil. In Espírito Santo, the situation is no different; recent data (IPEA/FBSP, 2023; IPEA/FBSP, 2024) indicate that 12 MP officers, both on and off-duty, were killed in the state between 2020 and 2024, which is a deeply concerning statistic.

Furthermore, scientific literature has demonstrated that public security officers are disproportionately exposed to work-related stress. For example, Pelegrini et al. (2018) analyzed perceptions of working conditions and occupational stress among 84 public security officers—both civil and military—within special operation units. Their study found that one in four officers experienced either low work demand with limited decision-making authority or high work demand with minimal decision-making power, making them more susceptible to illness.

It is recognized that stress can have varying impacts, some of which may be beneficial. Studies by Poletto et al. (2009) and Tricoli (2010) indicate that stress can be a positive factor, facilitating skill acquisition and the ability to overcome obstacles in situations requiring rapid reactions and decision-making. Conversely, stress can also be detrimental, causing significant harm to individuals’ physical, cognitive, psychological, and social well-being, as Ferreira (2009) and Tricoli (2010) explored.

Given these circumstances, it is essential to investigate variables that can enhance the survival prospects of this highly vulnerable group in confrontational scenarios. In this regard, Simas et al. (2022) demonstrated that the presence of stress symptoms diminishes the precision of firearm shooting, a factor that can significantly influence the accuracy of shots fired by police officers.

Thus, recognizing that the accuracy of firearm shooting is a fundamental factor for police survival given that highly effective shooting can prevent unjust aggression and increase the survival chances of security officers, as well as civilians directly or indirectly involved in armed confrontations.

Therefore, psychological and motor skill should be trained to perform under stress in actual operations (Pallavicini et al., 2016). According to several studies (Zueger et al., 2022; Brinkmann et al., 2022; Hoareau et al., 2021; Millegan et al., 2021). This study primarily aims to investigate the influence of stress levels on the accuracy of firearm shooting. Additionally, this study will also seek to explore, as a secondary objective, the influence of stress levels on other variables, such as handgrip strength (HGS), which is identified in the scientific as a factor that may impact the precision of firearm shooting (Marković et al., 2020; Muirhead et al., 2019). The initial hypothesis of this research is that the presence of stress symptoms may diminish the precision of firearm shooting, as suggested by the study conducted by Simas et al. (2022). Therefore, the objective of this study was to determine whether the presence of stress symptoms affects HGS and the accuracy of firearm shooting among MP officers.

## 2 Materials and methods

### 2.1 Demographics and sample selection

This research was approved by the Human Research Ethics Committee of the Federal University of Espírito Santo (No. 6.275.609/2023) and authorized by the Board of Education of the Military Police of Espírito Santo (PMES). Active-duty MP officers of both sexes, as well as cadets in their final year of the Military Police Officer Training Academy (CFO) at PMES, were invited to participate voluntarily in the study. Upon agreeing to participate, the military personnel signed an Informed Consent Form (ICF) by the standards set by Ordinance No. 466/2012 of the National Health Council.

Exclusion criteria included military personnel absent from work during the study period for any reason and those who did not sign the consent form. Additionally, participants who did not respond or did not complete all phases of data collection and analysis were excluded. After applying the inclusion and exclusion criteria, the final sample comprised 24 military personnel (22 men and 2 women), divided into two groups: those with stress symptoms (CE, *n* = 11) and those without (SE, *n* = 13).

### 2.2 Data collection methodology

The Military Police Officer Training Academy (MPOTA) serves as the foundational training program and prerequisite for entering the career of a Police Officer in the Military Police (Espírito Santo, 1978). Patrol officers’ primary responsibilities include supervising and supporting routine patrols across Espírito Santo. Cadets enrolled in the MPOTA engage in daily academic study and physical training for 3 years, after which they are designated as Officer Aspirants upon program completion.

Data collection for this study was conducted at the Military Police Academy of Espírito Santo (APM) during service days, based on the availability of the participants. On the first day, volunteers underwent anthropometric assessments and handgrip strength (HGS) measurements while wearing the standard physical training uniform. Additionally, they completed self-administered questionnaires addressing their stress and physical activity (PA) levels. The following day, data collection continued at the Academy’s shooting range. Participants were reacquainted with firearm shooting, dressed in the standard operational uniform, and equipped with personal protective gear (including ballistic vests, safety glasses, and hearing protection by regulations). In the afternoon, their firearm shooting proficiency was evaluated as part of the mandatory “Police Shooting III” course, a component of the CFO curriculum (Espírito Santo Conselho Estadual da Educação, 2016).

### 2.3 Assessed parameters

#### 2.3.1 Anthropometric measurements and body composition

For the anthropometric evaluation, participants’ body mass was measured using a Marte Científica digital scale (model LS200P, with a precision of 0.1 kg), and height was measured with a Cardiomed stadiometer (model WCE, with a precision of 0.1 cm).

Body mass index (BMI) was calculated by dividing body mass by the square of height. For analytical purposes, participants’ nutritional status was classified based on their BMI (kg/m^2^) as follows: normal (>18.5 and <25.0), overweight (≥25.0 and <30.0), and obese (≥30.0 and <40.0) (World Health Organization, 2000).

Body composition was estimated through a doubly indirect method by measuring skinfold thickness at the biceps, triceps, subscapular, and suprailiac regions using a Cescorf caliper (Mitutoyo model, with 0.1 mm precision). Body fat percentage (%BF) was calculated as proposed by Durnin and Womersley (1974). Participants were classified based on %BF as either non-obese (<25% for men and <30% for women) or obese (≥25% for men and ≥30% for women), as suggested by National Heart, Lung, and Blood Institute (1998).

#### 2.3.2 Stress levels assessment

Following recommendations of Lipp’s (2000), stress levels of participating MP were assessed using Lipp’s Inventory of Stress Symptoms (LISS), identifying the presence or absence of stress symptoms and provides a diagnosis based on physical and psychological indicators. It categorizes stress into four stages: alert, resistance, near exhaustion, and exhaustion. In summary, the instrument consists of three charts corresponding to different stages of stress. The first chart contains 15 items related to physical or psychological symptoms experienced in the last 24 h. The second chart includes 10 physical, and five psychological symptoms related to experiences in the past week. The third chart encompasses 12 physical, and 11 psychological symptoms experienced over the past month. This study focused solely on the presence or absence of stress symptoms. A military officer was classified as having stress symptoms if they marked four or more symptoms in Chart 1 of the LSSI, seven or more in Chart 2, or nine or more in Chart 3, according to Dalagnol (2022) and Rossetti et al. (2008).

#### 2.3.3 Physical activity assessment

Total weekly PA time was utilized to classify the PA level according to the International Physical Activity Questionnaire (IPAQ-short version), as applied in other studies (Ferraz et al., 2020; de Oliveira et al., 2023). The questionnaire explored the frequency and duration of physical activities, including walking and moderate and vigorous exercise. Military personnel were considered active or meeting physical activity (PA) recommendations if they achieved 150 min or more PA per week; those who did not meet this threshold were classified as inactive.

#### 2.3.4 Handgrid strength assessment

**Figure 1:**
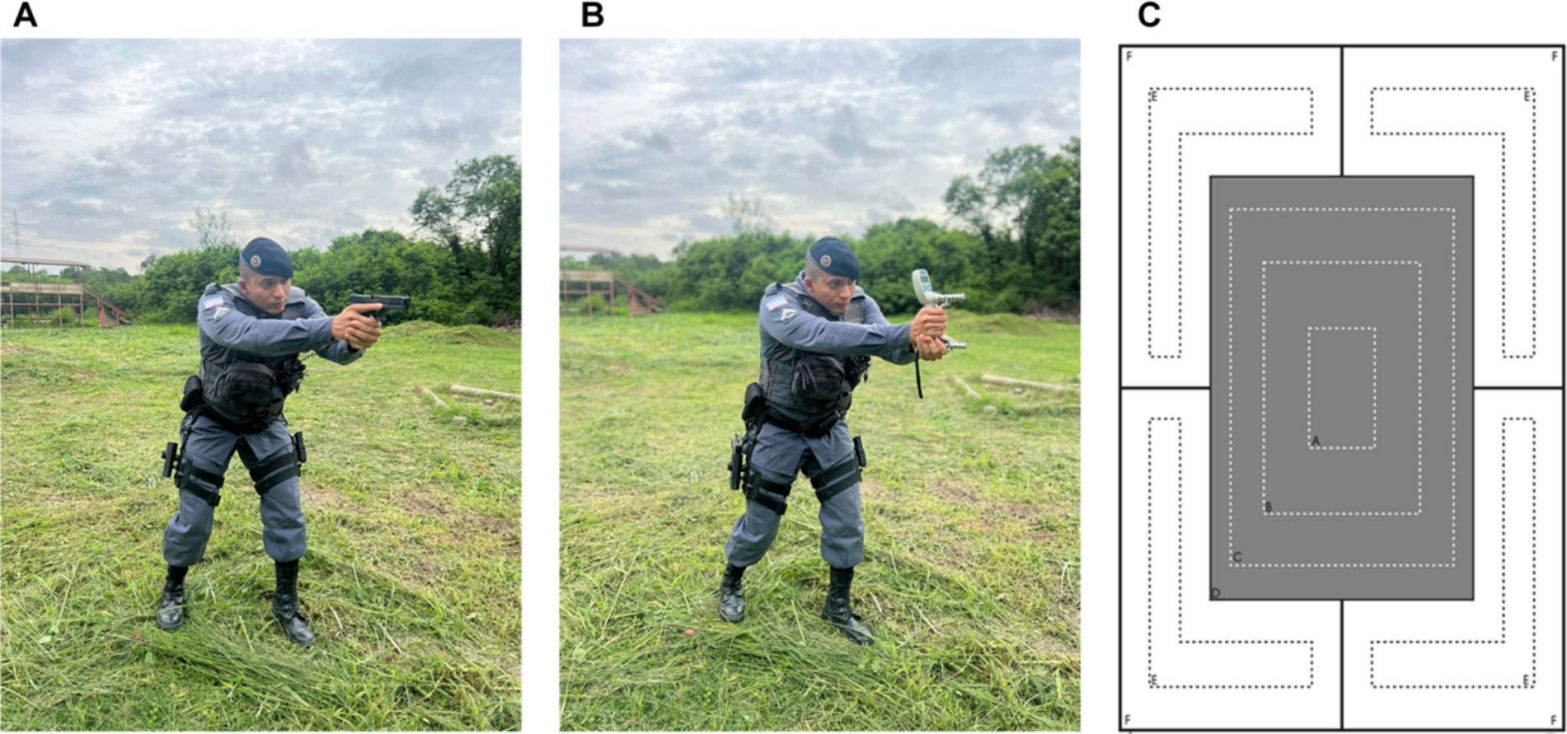
Images of police officer in shooting position with gun **(A)**, shooting position with handgrip equipment **(B)**, and target used on firearm shooting assessment **(C)**.

Handgrip strength (HGS) was assessed in kilograms (kg) using a Jamar handgrip dynamometer (Plus model) in two positions: the standard position (with the elbow at 90°) for both the dominant and non-dominant hands and the shooting position (Figures 1A,B) with the dominant hand as indicated by the volunteer.

In the standard position, participants were instructed to sit in a chair without armrests, maintaining a straight back, with knees bent at 90°, shoulders adducted in a neutral rotation, elbows flexed at 90°, and forearms in a semi-pronated position with wrists in a neutral alignment, allowing for extension up to 30°. The arm was elevated, and the dominant hand was positioned on the dynamometer, supported by the assessor, following the protocols established by Fess and Moran (1981). The same procedure was applied to the non-dominant hand. For the evaluation in the shooting position, participants were guided to assume the typical posture used during firearm shooting with the dominant hand.

Four measurements were taken in each position following an initial familiarization session with the dynamometer. Grip size adjustments were made as necessary. During the measurement, participants were instructed to squeeze the device with maximum strength for 10 s, with a 20-s rest interval between each measurement, as adapted from Kamimura and Ikuta (2001) and Trossman and Li (1989).

#### 2.3.5 Firearm shooting assessment

The “Defensive Shooting in Life Preservation (DSLP),” also known as the Giraldi Method, was developed by Colonel Nilson Giraldi to prepare military police MP officers for using firearms in service. This method emphasizes respect for the law and human rights and the preservation of the lives of officers and innocent bystanders. It focuses on conditioning and reinforcing positive reflexes through training that simulates operational realities (Giraldi, 1998).

Since 2002, the Giraldi Method has served as the official firearm instruction method of the Military Police of Espírito Santo (PMES), applied in all training, qualification, and improvement courses since its adoption (Polícia Militar do Espírito Santo, 2002). Since 2002, the Giraldi Method has served as the official firearm instruction method of the Military Police of Espírito Santo (PMES), applied in all training, qualification, and improvement courses since its adoption (Polícia Militar do Espírito Santo, 2002).

The “Basic Course Evaluation—0.40 S&W Pistol” was administered to assess shooting accuracy without external distractions or pressure. This test represents the initial stage of the Giraldi Method’s user training program. During the evaluation, participants donned operational uniforms and utilized the institution’s standard sidearm, the Glock G22 Gen 5 0.40 caliber pistol.

This assessment occurs after a training day focused on the fundamentals of shooting. Military personnel fired 10 shots at a target positioned 5 meters away from various positions (according to standard dynamics), aiming to strike the target’s central zone (gray zone), which measures 50×32 cm on a target measuring 80×54 cm (Figure 1C). Hits outside the gray zone (white zone) were not counted. The test commenced with participants shooting two rounds while standing, two rounds while kneeling, two rounds while crouching, two rounds while prone, and concluding with two rounds while standing again (Giraldi, 1998).

In addition to the number of hits on the target (total score, TS), the time taken to complete the task (T) was recorded. This allowed for calculating the shooting accuracy coefficient, derived from the ratio of hits to the time taken, multiplied by a factor of 50 (50 × TS/T). This calculation underscores the importance of the police officer’s reaction time, officer’s reaction time, reflected in their shots’ speed and accuracy. Consequently, an officer who accurately hit all targets quickly received a superior score than one who achieved all hits but took longer.

### 2.4 Statistical analysis

Quantitative variables are presented as means ± standard deviations. Following the assessment of data distribution normality using the Shapiro–Wilk test, comparisons between the conditions of military personnel in the stress symptom group (SE) and the no-stress symptom group (NSE) were performed. Absolute and relative typical error of handgrip strength assessment in shooting position was evaluated according to the model used by our laboratory (Bravin et al., 2024). The Student’s t-test was used for parametric variables, while the Mann–Whitney U test was employed for non-parametric variables. Furthermore, Pearson’s correlation test was applied to analyze the relationship between the shooting accuracy coefficient (50 × TS/T) and the variables that exhibited statistical differences between the CE and SE groups. The correlation strength was classified as follows: weak (0.10 ≤ r < 0.30), moderate (0.30 ≤ r < 0.60), and strong (0.60 ≤ r ≤ 1.00). The effect size was estimated using Hedges’ g, interpreted as small (0.20 ≤ ES < 0.50), moderate (0.50 ≤ ES < 0.80), and large (0.80 ≤ ES ≤ 1.00). Statistical analyses were conducted using GraphPad Prism software version 6.00 for Windows, with results presented as means ± standard deviations. A significant level of *p* < 0.05 was adopted for all statistical tests.

## 3 Results

Of the military personnel assessed, 11 individuals (45.8%) exhibited four or more stress symptoms in chart 1 or seven or more symptoms in chart 2 of Lipp’s Inventory of Stress Symptoms (LSSI), categorizing them in the alert or resistance phases. Notably, no participants reported nine or more symptoms in chart 3 of the LSSI. Statistical differences were found in physical (SE: 3.18 ± 0.87; NSE: 0.93 ± 0.96; MD: 2.24; 95%; CI: −3.007–−1.490; *p* < 0.0001) and psychological (SE: 2.18 ± 1.25; NSE: 0.66 ± 0.81; MD: 1.51; 95%; CI: −2.351–−0.679; *p* < 0.0010).

Table 1 summarizes the general characteristics of the sample. Statistical analysis revealed no significant differences in age, length of service, or body composition parameters between the stress symptom group (SE) and the no-stress symptom group (NSE).

**Table 1:** Demographic and physical attributes of military police officers.

Parameters	Overall	SS	NSS	DM (95% IC)	ES	*p*
Age (years)	31.13 ± 2.49	31.36 ± 2.66	30.92 ± 2.43	0.44 (−2.59–1.71)	0.17	0.678
Service (years)	7.71 ± 3.75	8.73 ± 3.20	6.85 ± 4.08	1.88 (−5.02–1.26)	0.50	0.161
Body mass (kg)	79.70 ± 10.98	77.41 ± 12.48	81.63 ± 9.62	4.22 (−5.13–15.58)	0.38	0.359
Height (m)	1.76 ± 0.08	1.73 ± 0.09	1.79 ± 0.06	0.06 (−0.00–0.12)	0.79	0.064
BMI (kg/m^2^)	25.58 ± 2.45	25.73 ± 2.80	25.46 ± 2.21	0.27 (−2.39–1.85)	0.10	0.798
%BF	12.90 ± 3.54	13.20 ± 3.21	12.60 ± 3.90	0.61 (−3.68–2.44)	0.17	0.494
FM (kg)	10.40 ± 3.48	10.30 ± 3.08	10.40 ± 3.92	0.14 (−2.87–3.17)	0.04	0.919
LBM (kg)	69.30 ± 9.40	67.10 ± 11.09	71.20 ± 7.66	4.07 (−3.89–12.04)	0.43	0.301

Values are expressed as mean ± SD of stress symptom group (SS) and the no-stress symptom group (NSS). BMI: Body Mass Index. %BF, Body Fat Percentage; FM, Fat Mass; LBM, Lean Body Mass.

Table 2 presents data on the sample’s average weekly physical activity duration, which is categorized into light physical activity (walking). moderate intensity. and vigorous intensity. No significant differences were observed between the SS and NSS groups in these categories.

**Table 2:** Weekly duration of physical activity among military police officers.

Parameters	Overall	SS	NSS	DM (95% IC)	ES	*p*
Walking	247.08 ± 333.53	88.64 ± 75.24	381.15 ± 407.45	292.50 (33.24–551.80)	0.95	0.02
Moderate PA	165.42 ± 151.46	136.82 ± 89.73	189.62 ± 189.41	52.80 (−76.69–182.30)	0.34	0.40
Vigorous PA	175.58 ± 201.28	165.91 ± 223.48	183.77 ± 189.40	17.86 (−156.80–192.50)	0.08	0.57
Total PA	588.08 ± 601.04	391.36 ± 259.46	754.54 ± 755.52	363.30 (−133.60–860.00)	0.63	0.14

Values are expressed as mean ± SD stress symptom group (SS) and the no-stress symptom group (NSS). PA: Physical Activity.

First, the reliability of handgrip strength in shooting positions was evaluated using two assessments on two different days. No differences were found between evaluations (1°: 42.68 ± 10.40, 2°: 42.62 ± 10.93 kg; mean difference: 0.05; 95% confidence interval: −1.205–1.090; *p* = 0.918). Significant linear (*r:* 0.96, *p* < 0.0001) and intraclass (*r:* 0.96, *p* < 0.0001) correlations were found between evaluations, with small absolute (0.11) and relative (0.27%) typical errors.

**Figure 2:**
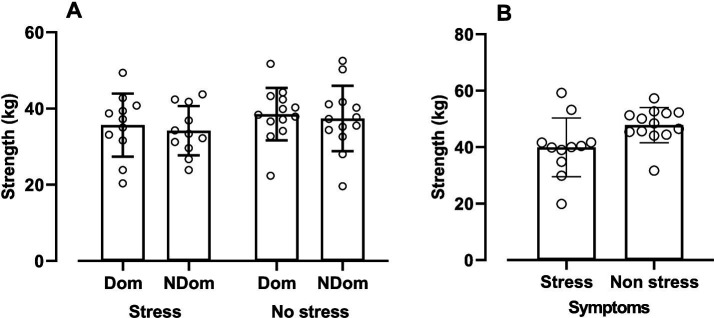
Values expressed as mean ± SD of military police officer with and without stress symptoms. **(A)** Handgrip strength in the dominant hand (Dom) and non-dominant hand (NDom). **(B)** Shooting position with the dominant hand.

Figure 2 illustrates handgrip strength in both the standard and shooting positions. In the standard position (Figure 1A), no significant differences in handgrip strength were found between the groups (*F*_1,48_ = 0.38; *p* = 0.53) or within the CE and SE groups (*F*_1,48_ = 0.46; *p* = 0.49). Furthermore, there were no differences in handgrip strength between the dominant and non-dominant hands within the CE group (DH: 35.66 ± 8.28 kg; NDH: 34.19 ± 6.47 kg; DM: 1.47; 95% CI: −5.21–2.27; ES: 0.17; *p* = 0.40) or the SE group (DH: 38.54 ± 6.88 kg; NDH: 37.39 ± 6.47 kg; DM: 1.14; 95% CI: −3.75–1.46; ES: 0.17; *p* = 0.25).

However, in the shooting position with the dominant hand (Figure 1B), a significant difference in handgrip strength was found between the CE and SE groups (CE: 39.96 ± 10.40 kg; SE: 47.06 ± 8.57 kg; DM: 7.86; 95% CI: 0.73–15.00; ES: 0.88; *p* = 0.03).

**Figure 3:**
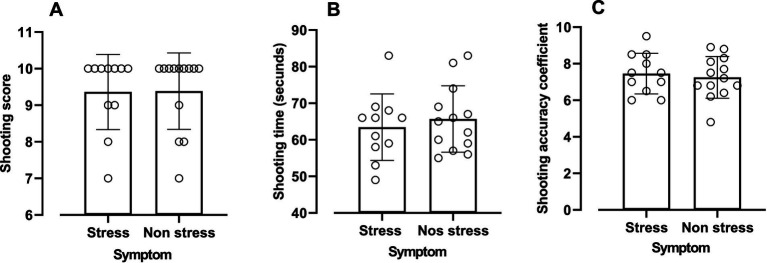
Values expressed as mean ± SD for shooting scores **(A)**, shooting time **(B)**, and shooting accuracy coefficient **(C)** for military police officers with and without stress symptoms.

Figure 3 presents the firearm shooting accuracy data for the CE and SE groups. No significant differences were identified between the groups in terms of score (CE: 9.36 ± 1.02 points; SE: 9.38 ± 1.05 points; DM: 0.02; 95% CI: −0.85–0.90; ES: 0.01; *p* = 0.96), time (CE: 63.45 ± 9.09 s; SE: 65.69 ± 9.08 s; DM: 2.23; 95% CI: −5.48–9.96; ES: 0.24; *p* = 0.55), or shooting accuracy coefficient (CE: 7.45 ± 1.10; SE: 7.25 ± 1.14 points; DM: 0.20; 95% CI: −1.15–0.75; ES: 0.17; *p* = 0.66).

**Figure 4:**
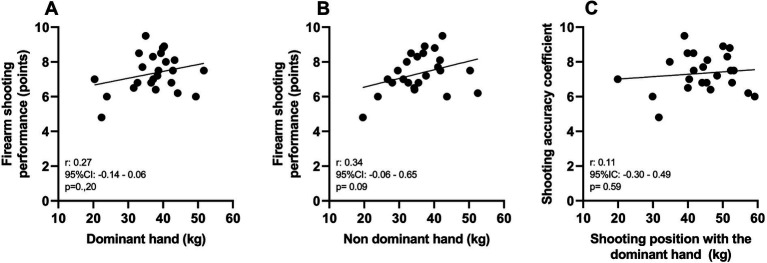
Correlation between the shooting accuracy coefficient and handgrip strength of the dominant hand **(A)**, non-dominant hand **(B)**, shooting position with dominant hand **(C)**.

**Figure 5:**
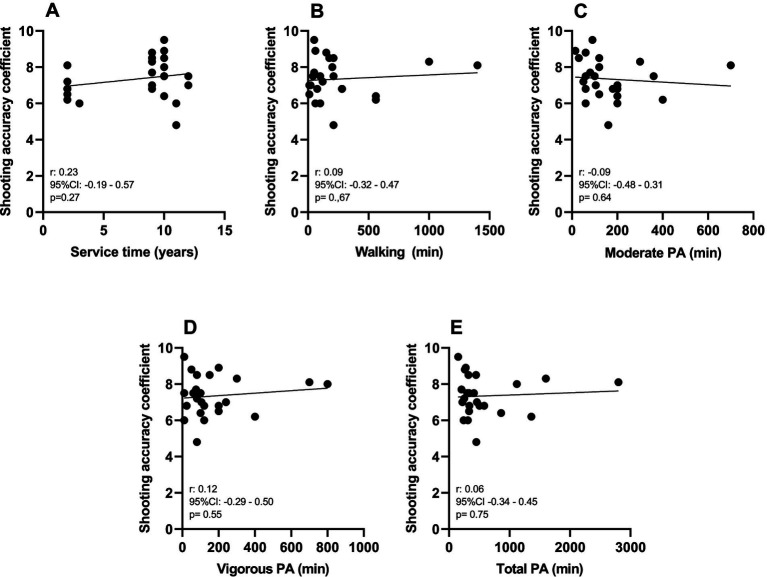
Correlation between the shooting accuracy coefficient and length of service **(A)**, walking physical activity time **(B)**, moderate physical activity time **(C)**, vigorous physical activity time **(D)**, and total weekly physical activity time **(E)**.

As illustrated in Figures 4, 5, no correlations were found between the shooting accuracy coefficient and the various handgrip strength measures (Figure 3) or with the length of service and different durations of weekly physical activity (Figure 4).

## 4 Discussion

Professional activity has been identified as a significant factor in an individual’s quality of life, as work constitutes one of humans’ primary activities (Souza Filho et al., 2015). In this sense, the routine of occupational activities can physically and emotionally strain workers. It negatively impacts their health and overall perception of quality of life.

Among occupational activities, MP work is recognized as particularly stressful, prompting numerous studies investigating this population from various perspectives. In this regard, Benevides-Pereira (2002) identifies military work as one of the professions most exposed to risks, with the health and well-being of individuals constantly under threat.

In this context, it is crucial to emphasize that the military personnel evaluated in this study were cadets from the Military Police Officer Training Academy (CFO). These individuals regularly face high demands and engage in routine physical training, complemented by rigorous psychological preparation, as they prepare to serve as law enforcement officers. Despite this background, a significant proportion of military personnel (45.8%) exhibited stress symptoms. This may be linked to the specific phase course phase they underwent while finalizing and presenting their thesis, completing operational internships, and concluding academic assessments. Previous research has similarly noted increased stress levels due to the police training course and reported heightened stress in a substantial portion of the group after 6 months of training (Sousa et al., 2020).

The literature consistently indicates that PA contributes to neuromuscular responses, enhances cardiorespiratory function, maintains body mass, and promotes overall well-being, reducing anxiety and depressive symptoms (de Mello et al., 2013; Wiles et al., 2012). Furthermore, engaging in PA can alleviate tension and mitigate the detrimental effects of stress (Souza et al., 2013). Several studies (Nascimento Júnior et al., 2012; Rueggueberg et al., 2012) have demonstrated an inverse association between levels of PA and stress levels among adults, the older people, and younger populations. Consequently, it is plausible that the relatively low incidence of stress symptoms observed in this study may have been influenced by the regular physical activities incorporated into the CFO curriculum. However, further research is needed to elucidate these findings.

As with previous research (Brandão, 2024; Dutra et al., 2023), the sample in this study was predominantly male. This gender prevalence may be directly linked to the recruitment processes within the MP, as specific competitive examination announcements explicitly indicate that the number of vacancies available for women is lower than that allocated to men (Lima et al., 2015). However, it is noteworthy that the admission notice for the Military Police Officer Training Academy at PMES does not differentiate or limit vacancies based on the candidate’s gender, thereby preventing the attribution of this predominance to Public Administration practices. This is just one of the limitations of our study that we need to be aware of when interpreting the results.

Despite the average body mass index of 25.58 ± 2.45 kg/m^2^ categorizing the military personnel as overweight, the recorded body fat percentage of 12.90 ± 3.54% is considered excellent for both groups (Table 1). This finding contrasts with other studies (Itacarambi et al., 2019; Santos et al., 2021), which identified a high prevalence of elevated body mass among military police officers. Therefore, it is recommended that future assessments of obesity in military personnel from Espírito Santo not solely rely on BMI but also incorporate additional parameters, such as body fat percentage, to provide a more comprehensive evaluation.

Although HGS is a commonly utilized assessment in other countries (Brown et al., 2021; Orr et al., 2021; Marković et al., 2020; Muirhead et al., 2019; Orr et al., 2017), its usage among Brazilian MP officers is non-existent; therefore, studies are limited to non-existent. To illustrate the significance of this data, Orr et al. (2017) demonstrated a positive association between HGS and performance in police tasks, indicating that recruits with underdeveloped HGS face a higher risk of failing to execute their occupational duties.

In this study, no alterations were observed in the development of HGS among military personnel exhibiting symptoms of stress, a factor that may have influenced firearm shooting accuracy. Several studies investigating the relationship between shooting accuracy and HGS (Brown et al., 2021; Orr et al., 2021; Marković et al., 2020; Muirhead et al., 2019) suggest that further research is warranted to deepen the understanding of these parameters.

However, Orr et al. (2021) demonstrated that hand size and HGS significantly impacted firearm shooting accuracy, suggesting that hand size may play an essential role in this context. Additionally, the researchers propose the existence of a V-shaped curve among HGS. Accuracy, arm span, and aim, with the standard grip size of the firearm acting as an intervening factor. Similarly, Brown et al. (2021) found that HGS significantly affected the shooting capability of police officers, indicating that those with lower grip strength achieved fewer hits while using a firearm. Furthermore, the authors demonstrated that a grip strength development ranging from 80 to 125 pounds (approximately 36.3 to 56.7 kg) was necessary to attain approximately 85 to 95% shooting accuracy.

In the present study, HGS was found to be near the lower threshold suggested by Brown et al. (2021). This finding warrants further studies investigating this parameter within the Brazilian population. Incorporating manual grip strength training into military physical training programs is also advisable to enhance police shooting accuracy. Additionally, the development of greater HGS may facilitate the trigger pull of firearms, as overcoming the resistance could become more accessible.

Marković et al. (2020) and Muirhead et al. (2019) suggest a correlation between shooting accuracy and HGS. However, they note that hand size and the weapons’ dimensions used in the assessment may influence outcomes. In contrast, the current research did not find a correlation between HGS and indicators of shooting accuracy. Therefore, it is essential to conduct further research using diverse scenarios, incorporating various types and weights of firearms under different shooting conditions. Moreover, studying other groups of Brazilian military personnel may help elucidate these findings.

From the PA level perspective, all volunteers reported weekly PA exceeding the World Health Organization’s (World Health Organization, 2020) minimum recommendation of 150 min. The overall group averaged 588.08 ± 601.04 min of weekly PA, categorizing them as active or very active. However, no statistical differences were observed between the SS and NSS groups regarding total reported weekly PA durations; variations were noted in the time spent walking.

One potential explanation for the high level of PA observed in the studied sample is the regular practice of physical training during work hours, a common practice among active police officers in training programs (Boçon, 2015). Increased levels of PA are well-established as significant for promoting health and positively influencing the reduction of occupational stress factors among police officers. Furthermore, regular PA is known to alleviate anxiety and depression, enhance cognitive functions, and strengthen muscles, thereby contributing to the effective execution of specific missions (Vancini et al., 2018).

The scientific literature evaluating firearm shooting parameters conducted by Brazilian police officers is scarce (Nascimento Neto, 2015; Nascimento Neto et al., 2017). In addition, researchers address these scenarios, which hinder the precise assessment of outcomes. For instance, Nascimento Neto et al. (2017) evaluated the shooting accuracy of MP officers following a physical effort protocol, while Nascimento Neto (2015) investigated the effects of transcranial direct current stimulation on their shooting accuracy.

In the current research, no differences in shooting accuracy were observed between the groups with and without symptoms of stress. This finding contrasts with the results of Simas et al. (2022), which indicated that stress can directly affect firearm shooting accuracy. This divergence may be attributed to the military personnel’s training before the shooting evaluation, which could have mitigated potential adverse performance outcomes.

Moreover, it is essential to note that most security agents were already state military personnel before entering the Military Police Officer Training Academy, particularly given the group’s average service time of over 7 years, while the CFO is completed in 3 years. Thus, it is essential to consider that these military personnel already had shooting experience, which may have significantly influenced their shooting accuracy. Nevertheless, no correlation was found between length of service and shooting accuracy. Therefore, it can be inferred that by the end of the CFO, all Officer Cadets, on average, achieved a normalized shooting accuracy coefficient according to the current procedures, indicating that prior length of service in the police department did not meaningfully impact accuracy outcomes.

The findings of this study indicate that there were no differences in the total scores and shooting accuracy coefficients between military personnel with and without symptoms of stress during firearm shooting. Therefore, MPs should conduct regular training exercises to ensure continuous improvement in this skill.

Several limitations were evident in the current research, which restricts its generalizability. Among the limiting factors, the following can be highlighted: the sample size and predominium (composed of men); the assessment of stress levels and PA conducted through self-administered questionnaires, which may have introduced significant variability in the data; the analysis focused on military personnel not primarily engaged in operational activities; the evaluation of shooting accuracy within an educational setting, and the implementation of shooting training immediately before the assessment.

Moreover, the future of shooting evaluations lies in incorporating diverse types of firearms, which could significantly influence personality traits such as neuroticism and resilience, as well as physiological stress indicators on military and police performance, which could clarify our findings. This approach will provide a more comprehensive assessment of shooting accuracy and performance. It is crucial to conduct shooting evaluations without subjecting military personnel to training beforehand. Incorporating personnel directly engaged in operational activities and those with varying lengths of service is also essential. Furthermore, assessing specialized groups under conditions of physical and emotional fatigue that differ from those of ordinary troops could facilitate more robust analyses.

In conclusion, the data from the current research suggest that the presence of stress symptoms, although associated with varying levels of HGS development in the shooting position, did not change firearm shooting accuracy among cadets in the Military Police Officer Training Course.

## Data Availability

The raw data supporting the conclusions of this article will be made available by the authors under reasonable request.
